# Lecithin Characteristics from Niche Oils

**DOI:** 10.3390/molecules31132274

**Published:** 2026-06-29

**Authors:** Joanna Harasym, Weronika Wójcik

**Affiliations:** 1 Department of Biotechnology and Food Analysis, Wroclaw University of Economics and Business, Komandorska 118/120, 53-345 Wroclaw, Poland; 2 Adaptive Food Systems Accelerator-Science Centre, Wroclaw University of Economics and Business, 53-345 Wroclaw, Poland; 3 Faculty of Production Engineering, Wroclaw University of Economics and Business, 53-345 Wroclaw, Poland; 190982@student.ue.wroc.pl

**Keywords:** lecithin, phospholipids, phosphatidylcholine, vegetable oils, hemp, flax, camelina, pumpkin seed oil, degumming, oil bodies

## Abstract

Plant lecithins are complex mixtures of phosphatidylcholine (PC), phosphatidylethanolamine (PE), phosphatidylinositol (PI), phosphatidic acid (PA) and lysophospholipids. They are increasingly demanded as natural, non-allergenic emulsifiers and as nutraceutical carriers. Quantitative data on the phospholipid (PL) fraction of less-commodity oilseeds, however, remain dispersed. This review compares the PL composition, processing-dependent extractability, and functional behavior of six oils: hemp (*Cannabis sativa* L.), sunflower (*Helianthus annuus* L.), corn/maize (*Zea mays* L., *germ*), pumpkin (*Cucurbita* spp.), flax/linseed (*Linum usitatissimum* L.), and camelina (*Camelina sativa* L.). Across the six oils, total PL content varies by more than an order of magnitude (c.a. 0.25% in camelina oil bodies; >1% in solvent-extracted hemp and c.a. 0.5–1.0% in Styrian pumpkin oil), and PC fraction ranges from 30% (hemp seed tissue) to 56% (rapeseed-comparable sunflower). Flax stands out for both an exceptionally high PE share (22.7%) and a polyunsaturated fatty acid (PUFA) content in the PL fraction (53.3%) that exceeds rapeseed, sunflower and soy by an order of magnitude. We integrate recent extraction (cold pressing, supercritical CO_2_, microwave-assisted, aqueous enzymatic) and degumming data (water, acid, enzymatic with phospholipases A1/A2/C) with functional evidence in emulsions, oil bodies, and bioactive delivery.

## 1. Introduction

**Figure 1:**
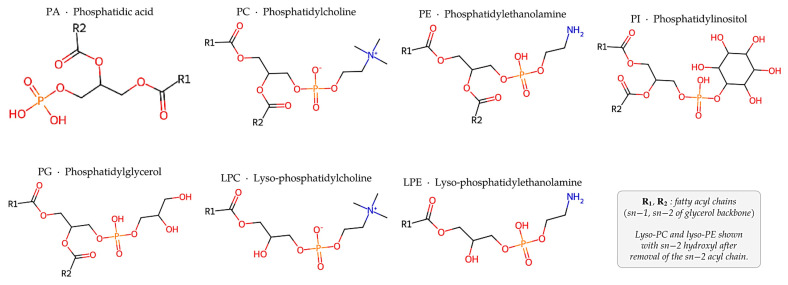
Generic structures of the principal plant phospholipid classes. PA—phosphatidic acid (free phosphate); PC—phosphatidylcholine (choline-phosphate; zwitterionic at physiological pH); PE—phosphatidylethanolamine (ethanolamine-phosphate); PI—phosphatidylinositol (myo-inositol-phosphate); PG—phosphatidylglycerol (glycerol-phosphate). Lyso-PC (LPC) and lyso-PE (LPE) are shown with a free sn-2 hydroxyl after enzymatic or chemical removal of the sn-2 acyl chain. Black—carbon, red—oxygen, orange—phosphorus, blue—nitrogen.

Lecithins are commercial-grade mixtures of glycerophospholipids recovered as a by-product of vegetable-oil refining and are dominated by phosphatidylcholine (PC), phosphatidylethanolamine (PE), phosphatidylinositol (PI), phosphatidic acid (PA), and minor lysophospholipids (LPC, LPE) [1,2]. The phosphate-bearing head group on a 1,2-diacyl-sn-glycerol backbone confers amphiphilicity, hydrogen-bonding capacity at the oil-water interface, and affinity for lipophilic micronutrients—properties that underlie lecithin use food emulsifier, liposomal carrier, and nutraceutical (Figure 1) [2,3]. Recoverable lecithin yield differs roughly tenfold between sources, which decides whether an oil is a viable commercial feedstock or only a specialty one.

**Figure 2:**
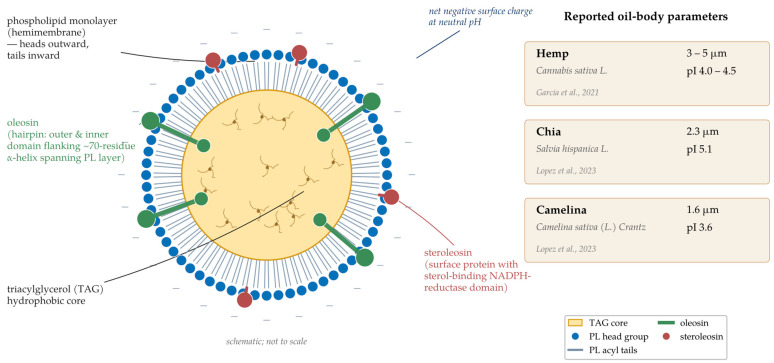
Schematic cross-section of a plant seed oil body (oleosome) and reported parameters for hemp, chia and camelina. Right panel: reported mean Sauter diameters and isoelectric points (pI) of oil bodies from hemp [4], chia and camelina [5]. Schematic; not to scale.

In the seed itself, PLs play a structural rather than a storage role—they form the monolayer that coats oil bodies (oleosomes) and surrounds organellar membranes, while triacylglycerols (TAGs) accumulate inside oil bodies as the energy store mobilized on germination [4,5]. Lipidomic profiling of *Cannabis sativa* has confirmed this division; in developing hemp seeds PC accounts for 30.6% of the total lipid pool but in root tissue the same class reaches 40.4% of total lipids, with PA additionally rising to 21.9%—reflecting the contrasting biosynthetic priorities of storage versus active membrane [6]. In the oleosomes (plant oil bodies) the triacylglycerol (TAG) hydrophobic core (Figure 2—yellow) is surrounded by a phospholipid hemimembrane in which the polar head groups (Figure 2—blue) face outward and the acyl chains (Figure 2—gray blue) extend inward toward the TAG core. Two surface-associated protein families are fused in the monolayer: oleosins (Figure 2—green), which adopt a hairpin topology with a c.a. 70-residue membrane-spanning α-helix flanked by N- and C-terminal hydrophilic domains, and steroleosins (Figure 2—red), which carry a sterol-binding NADPH-reductase domain on the cytoplasmic face. Negative signs in the outer halo indicate net negative surface charge at neutral pH, arising from the phosphate head groups and the surface protein carboxylates.

Soybean lecithin currently supplies most of the world market (65–75% of total PL in deoiled products), but soy allergenicity, the predominantly GM origin of the soybean supply, and the demand for clean-label ingredients are driving interest in alternative plant lecithins [2,7]. The six oils examined here cover the principal alternatives—hemp and flax for their omega-3-rich PL fractions; sunflower as the established non-GM lecithin source with industrial-scale processing infrastructure [8]; corn germ as a high-volume by-product of dry milling [9,10]; pumpkin (particularly *Cucurbita pepo* var. *styriaca*) as a specialty oil with the highest reported PL content among the six [11,12]; and camelina as the emerging Brassicaceae oilseed amenable to genetic engineering for long-chain PUFA accumulation [5,13,14].

The allergenicity of an edible oil or its lecithin is protein-mediated rather than lipid-mediated: it arises from residual seed storage and defense proteins (for soy, the Gly m group of allergens) that are co-extracted with the lipid, not from the triacylglycerol or phospholipid fractions themselves. Fully refined oils are largely deproteinated and are generally tolerated even by sensitized individuals, whereas cold-pressed and unrefined oils and crude lecithins recovered at the degumming stage retain more residual protein and therefore greater allergenic potential [2,7,8].

Therefore two consequences follow for lecithin sourcing: 1—the demand for ‘non-allergenic’ emulsifiers is, in practice, a demand for sources outside the major regulated allergen groups and for processing that minimizes protein carry-over; and 2—among the alternatives, sunflower and canola are valued specifically as non-GM, allergen-free, clean-label sources.

This review discusses the quantitative PL data available for these six oils and links composition to processing (cold pressing, supercritical CO_2_, microwave pretreatment, aqueous enzymatic extraction), refining (water, acid, enzymatic degumming, bleaching), and functional behavior (emulsion stability, oxidative kinetics, sn-2 positional fatty acid distribution), compiling (1) the botanical and gross seed-composition baseline, (2) the quantitative PL composition, and (3) the principal processing parameters and their effect on the PL fraction.

## 2. Botanical Background and Seed Composition

**Figure 3:**
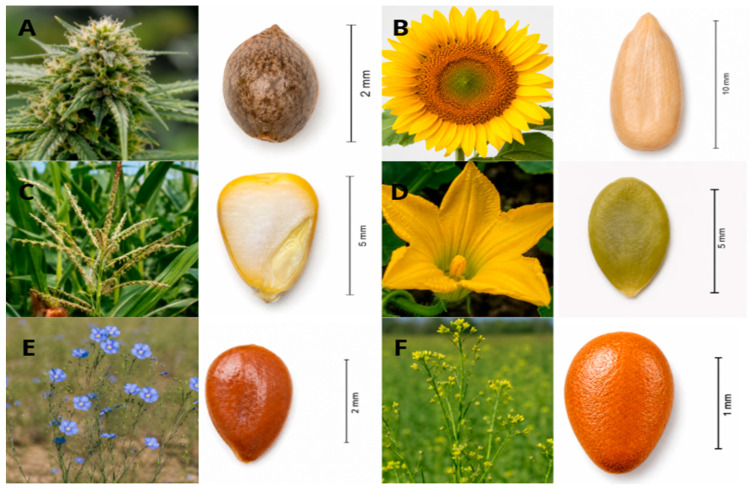
(**A**) *Cannabis sativa* L. (Cannabaceae); (**B**) *Helianthus annuus* L. (Asteraceae); (**C**) *Zea mays* L. (Poaceae)—kernel cross-section with the germ region indicated; (**D**) *Cucurbita pepo* L. var. *styriaca* (Cucurbitaceae), shown as the hull-less seed characteristic of the Styrian cultivar; (**E**) *Linum usitatissimum* L. (Linaceae); (**F**) *Camelina sativa* (L.) Crantz (Brassicaceae).

The six oilseeds chosen—hemp, sunflower, corn, pumpkin, flax and camelina—together represent the principal non-soy plant sources from which alternative lecithins can be recovered at industrial scale or in specialty markets. Being the most frequently occurring niche oils, they come from six distinct plant families and four geographic-evolutionary origins, ranging from the temperate-Eurasian *Cannabis sativa*, *Linum usitatissimum* and *Camelina sativa* to the American-domesticated *Helianthus annuus*, *Zea mays* and *Cucurbita* spp. (Figure 3). This phylogenetic and biogeographic diversity translates directly into the marked differences in oil content, protein content and dominant fatty acid profile summarized in Table 1.

The botanical background strongly impacts oil and protein content and the dominant fatty acid profile (Table 1). Their phylogenetic spread—Cannabaceae (hemp), Asteraceae (sunflower), Poaceae (corn), Cucurbitaceae (pumpkin), Linaceae (flax), and Brassicaceae (camelina)—drives a corresponding heterogeneity in oil-body architecture, PL biosynthesis, and storage-lipid composition.

Hemp seeds typically contain 25–35% lipids with linoleic acid (LA, 18:2 n-6) consistently above 50% of total fatty acids and α-linolenic acid (ALA, 18:3 n-3) at 16–20%, giving a near-ideal n-6/n-3 ratio of 2.5–3.4 [6,15]. Sunflower seeds contain 38–50% oil; the standard linoleic-type cultivars carry 55–70% LA, while high-oleic NuSun-type cultivars exceed 80% oleic acid and the high-stearic types accumulate up to 25% stearic acid, with corresponding consequences for PL acyl composition [1,8]. Maize germ—the embryo-rich fraction separated during wet or dry milling—contains 40–55% oil rich in LA (50–60%) and oleic acid (25–35%); the germ is the principal source of corn-oil PL, while pericarp and endosperm contain quantitatively distinct PL class distributions [9].

Pumpkin seeds (*Cucurbita pepo* L., *C. maxima* Duch., *C. moschata* Duch.) contain 40–54% oil; cold-pressed *C. pepo* var. *styriaca* (Styrian) oil shows ΣPUFA of 53.6–53.7% (predominantly LA), ΣMUFA 24–33% (oleic acid), and ALA generally < 0.5% [11,12,16,17]. Flax accumulates 35–45% oil with 35–55% ALA and an n-6/n-3 ratio close to 1:3—the highest land-plant ALA content among common oilseeds and the driver of flax PL’s unique fatty acid signature [3,18]. Camelina contains 27–49% oil and 24–31% protein; the standard cultivar profile is ALA 30–40%, oleic 15–20%, LA 15–20%, and the genus-specific 11-eicosenoic (20:1) and erucic (22:1) acids at 13–15% combined [5,19]. CRISPR-Cas9 inactivation of the *FAE1* elongase locus raised seed ALA from 36.9% (wild type) to 47.3% (*fae1*), while the DHA1×*fae1* line carrying transgenic algal desaturases/elongases accumulated 33.0% total C20^+^ n-3 fatty acids—a level comparable with refined marine oils [14].

Taken together, the six seeds converge on a broadly similar oil content (≈25–55% *w*/*w*) but diverge sharply in dominant fatty acid, splitting into an LA-dominated group (hemp, sunflower, corn, pumpkin) and an ALA-dominated group (flax, camelina)—a divergence that propagates directly into their phospholipid acyl signatures.

## 3. Total Phospholipid Content and Profile

Crude vegetable oils contain 1–3% total phospholipids; commercial deoiled lecithins concentrate this to 65–75% PL on a dry basis [2]; deoiling concentrates the PL roughly 20–30-fold—the step that turns a refining by-product into a functional ingredient. Across the six oils reviewed, the absolute PL content in the crude oil spans almost a tenfold range, and PC consistently dominates the class distribution except in maize endosperm, where PE is the major class [9].

Soybean lecithin remains the high-volume commodity standard: at PC ~47% it stabilizes oil-in-water emulsions chiefly through phosphatidylcholine and PC-protein complexes, but it carries allergen and predominantly GM-origin concerns. Rapeseed/canola lecithin (PC ~56%) is non-GM, low in protein-borne allergens and better suited to clean-label use, yet—like soy—it is PUFA-poor in the PL fraction. Against these two benchmarks, the value of the six niche oils lies in distinctive functionalities rather than in higher PL yield: the omega-3-bearing PL of flax (PE 22.7%, PL-PUFA 53.3%), the co-extracted Δ^7^-sterol/squalene/γ-tocopherol matrix of pumpkin, and the native oil-body delivery format of camelina [2,3,7,8]. Crude soy and sunflower lecithins give larger emulsion droplets (~0.62–0.64 µm) than PC-enriched fractions (0.391 µm), illustrating that the functional gap is governed by PL-class composition rather than oil identity [8].

**Figure 4:**
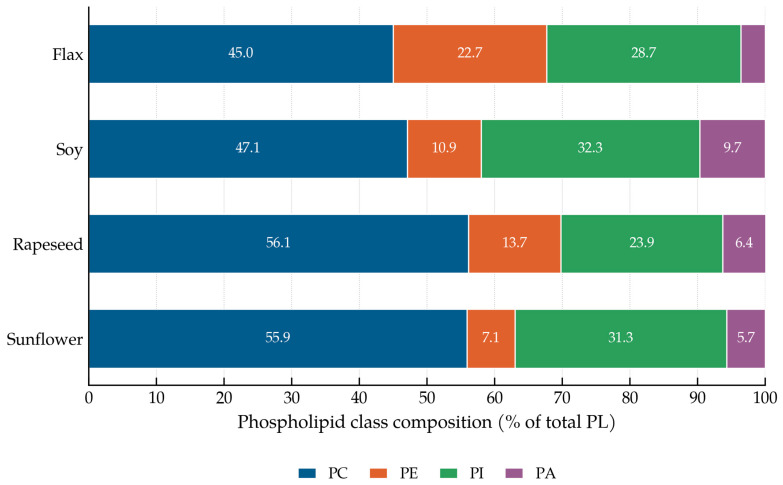
Phospholipid class distribution of four commercial plant lecithins (stacked horizontal bar chart, % of total PL) [3]. PC—phosphatidylcholine; PE—phosphatidylethanolamine; PI—phosphatidylinositol; PA—phosphatidic acid. Numerical values for each segment are shown inside the bars where segment width allows.

Quantitative class distributions are summarized in Table 2 and Figure 4.

### 3.1. Hemp

Lipidomic profiling of *Cannabis sativa* tissues distinguishes the PL fraction of developing seeds, leaves, and roots [6]. In developing seed, PC reaches 30.6% of total lipids while galactolipids (MGDG/DGDG—mono/digalactosyldiacylglycerol) collectively account for 44.6% (MGDG alone is 68% of the galactolipid pool); lysophospholipids account for 1.3%. In root tissue, PC becomes the dominant class at 40.4% of total lipids with PA second at 21.9%. The dominant molecular species in both MGDG and DGDG of hemp tissues is C36:6 (i.e., two 18:3 acyl chains), confirming *C. sativa* as a typical 18:3 plant in which membrane lipids are biosynthesized by the eukaryotic (ER-derived) pathway [6]. Quantitatively, crude hempseed oil is characterized by chlorophyll concentrations of 11,400 ppb (solvent-extracted) and 17,500 ppb (mechanically pressed)—the highest among the six oils—and by extraction-method-dependent PL load, with mechanically pressed crude oil carrying lower PL than hexane-extracted oil [20,21]. The highest pigment load of the six, which sets the heaviest bleaching demand and the largest accompanying loss of polar antioxidants.

### 3.2. Sunflower

Crude sunflower oil contains moderate PL levels relative to the other commodity oils; water degumming alone is insufficient to bring residual phosphorus below the 10 mg/kg, the regulatory ceiling below which an oil can be physically refined without alkali neutralization, so a secondary acid or enzymatic step is standard [1]. The PL class profile, when analyzed by HPLC in directly comparable conditions, is PC 56.1%, PE 13.7%, PI 23.9%, PA 6.4% (rapeseed-style sunflower lecithin reference); a separate sunflower fraction analyzed under the same protocol gave PC 55.9%, PE 7.1%, PI 31.3%, PA 5.7% [3]. Recent work fractionating sunflower crude lecithin into PC-enriched products has shown that increasing PC content reduces emulsion oil-droplet size to 0.391 µm and improves ice-cream microstructural stability, with overrun rising from 22.7% to 46.2% across the PC enrichment series [8].

### 3.3. Corn (Maize) Germ

Glycerophospholipid distribution in corn kernels is strongly tissue-specific [9]. In germ and pericarp fractions, PC is the dominant class at 51.4–70.6% of total glycerophospholipids, followed by PI (11.3–25.1%) and PE (8.4–12.6%). In the endosperm fraction the order inverts: PE becomes dominant at 41.4–48.5%, followed by PC (30.2–33.4%) and PI (13.2–14.4%) meaning commercial corn lecithin, drawn from the germ, is a high-PC product; whole-kernel figures would understate its PC content. The germ PC pool is enriched in molecular species containing PUFAs (predominantly PC-C18:2/18:1, *m*/*z* 768), whereas pericarp and endosperm PC pools are enriched in monounsaturated species (PC-C18:1/18:1, *m*/*z* 772). Commercial corn-oil lecithin is recovered from the germ fraction and therefore reflects the high-PC, PUFA-rich germ profile [9,10]. Corn-fiber gum, a co-product of wet milling, contains 0.24–0.43% bound lipids (mainly palmitic and oleic free fatty acids and sterol esters) that contribute to its emulsifying capacity at oil-water interfaces [22].

### 3.4. Pumpkin

Pumpkin seed oil (PSO) is the most PL-rich oil among the six on a wt.% basis: Styrian *C. pepo* var. *styriaca* oils show a total PL of 0.5–1.04 wt.% (determined as PA) with no significant correlation between seed phosphorus content (211 mg/g dry seed) and oil PL concentration [11], which shows total-P is not a usable approximation for lecithin yield in *Cucurbita.* Class-resolved data for *Cucurbita* PL is, however, sparse. The phytosterol fraction of pumpkin oil is dominated by Δ^7^-sterols (spinasterol, Δ^7^-stigmastenol, Δ^7^-avenasterol) rather than the more common Δ^5^-sterols of other plant oils, with β-sitosterol present only as a minor component [11,17]. Total phytosterols are reported at 718–898 mg/100 g oil (Serbian *Cucurbita*) and 782–805 mg/100 g (Anatolian *C*. *pepo*), squalene at 583–747 and 591–633 mg/100 g respectively, and γ-tocopherol consistently dominates the tocol pool [12,16,17]. The unusually high concomitant PL + Δ^7^-sterol + squalene + γ-tocopherol content positions pumpkin oil as a co-extracted nutraceutical matrix in which PL behavior cannot be considered in isolation from these lipophilic antioxidants.

### 3.5. Flax/Linseed

Flax lecithin produced commercially in Belarus has been characterized by HPLC against soy, rapeseed, and sunflower lecithins under identical conditions [3]. Flax PL is composed of PC—45.0%, PI—28.7%, PE—22.7%, and PA—3.6%—broadly comparable in PC content to soy (47.1%) but lower than rapeseed and sunflower (≈56%) and distinguished by an exceptionally high PE share (22.7%, against 7.1–10.9% in the comparator oils). The PA fraction (3.6%) is also markedly lower than in the comparators (5.7–9.7%), consistent with a less polar overall flax PL profile. The functional consequence is direct: PE-enriched PL mixtures form mixed micelles and bilayer-rim domains that influence interfacial behavior differently from PC-only mixtures, and PE preferentially carries DHA and arachidonate in mammalian neural membranes—making flax PL a candidate substrate for omega-3-targeted PE delivery [2,3].

### 3.6. Camelina

Class-resolved PL data for crude camelina oil is not available in the published literature. The available quantitative data comes from aqueous-extracted camelina oil bodies, in which PLs form the interfacial monolayer surrounding the TAG core [5]. These oil bodies have a mean Sauter diameter of 1.6 µm—smaller than the 3–5 µm of hemp oil bodies [4] and PLs account for 0.26% of total oil-body lipids, comparable to chia (0.23%), but a smaller droplet and lower isoelectric point than hemp—camelina oil bodies stay stable to lower pH but flocculate in mildly acidic foods. The associated β-sitosterol load is 2674 mg/kg oil and γ-tocopherol 670 mg/kg oil, with phenolic rutin also recovered in the aqueous phase. Camelina PL biology is regulated in part by phosphatidylcholine:diacylglycerol cholinephosphotransferase (PDCT), which interconverts PC and DAG and is therefore central to the changing of PUFAs from PC into TAG storage [13]. Recombinant *CsPDCT* in yeast microsomes did not discriminate between di-oleoyl, di-linoleoyl, and di-linolenoyl substrates and accepted erucoyl-DAG efficiently—explaining how erucic acid (22:1) can be assembled into camelina TAGs without proportionate PC pool enrichment. Under field phosphate deficiency, the camelina leaf PL:galactolipid ratio drops from 30% to 5% (indicating that low-phosphate cultivation raises oil yield but erodes the very PL fraction from which the lecithin is recovered), total seed oil rises by ~25%, and the FA profile shifts toward 18:1 and 20:1 monoenes at the expense of 18:3 [19]—a remodeling that has direct implications for camelina lecithin yield under marginal agronomic conditions.

Taken together, the six oils separate into a high-PC interfacial group (sunflower, corn germ) and a functionally specialized group defined by head-group or co-extract chemistry (flax PE, pumpkin Δ^7^-sterols, camelina oil bodies), with hemp intermediate.

## 4. Fatty Acid Composition of Phospholipids

The functional ranking of these lecithins follows from PL head-group geometry. Phosphatidylcholine is near-cylindrical (spontaneous curvature ≈ 0) and therefore forms stable lamellar interfacial films that give the smallest, most uniform droplets—the basis of soy/sunflower performance in O/W emulsions. Phosphatidylethanolamine is conical (negative curvature) and a poor stand-alone emulsifier, but this same geometry favors hexagonal phases and makes PE an efficient carrier of long-chain omega-3 acyl chains—the basis of flax PL’s nutritional, rather than purely interfacial, advantage. Charged classes (PI, PA) add electrostatic stabilization but are pH- and cation-sensitive. Superiority is thus oil-specific and application-specific, not absolute.

The acyl chains of PLs differ systematically from those of co-occurring TAGs in the same seed. The general rule, established by lipidomics across multiple species, is that PUFAs are over-represented in PLs in species whose membrane biosynthesis is dominated by the eukaryotic (ER) pathway but excluded from PLs in species that rely on prokaryotic-pathway (chloroplastic) PL assembly with saturated/MUFA acyl chains [2,6]. Among the six oils, flax exemplifies the extreme PUFA-incorporating phenotype, while sunflower and soy occupy the PUFA-excluding end [3].

### 4.1. Hemp

Hemp galactolipids and PLs are dominated by C36:6 molecular species (88% of MGDG in leaf), consistent with two 18:3 acyl chains and confirming hemp as a typical 18:3 plant [6]. In developing seed and seed-derived oil, LA accounts for ≈55% of TFA and ALA for 16–20% [15,23], and PLs in the seed reflect this composition with C18:2/C18:3 acyl combinations predominant. Hemp seed by-products (cake, hulls) retain 13–17% residual lipid, of which >97% are TAGs and a smaller PL fraction is co-extracted [23]. The dominant TAG molecular species in seed and by-product oils are 18:3/18:2/18:2, 18:3/18:2/18:1, and 18:3/18:3/18:2, indicating preferential pairing of 18:3 with 18:2 at glycerol positions and a corresponding under-representation of palmitic and stearic chains in the PL pool.

### 4.2. Sunflower

Sunflower PLs are strikingly low in PUFA: only 2–4% of total PL acyl chains are polyunsaturated, against 60–70% PUFA in the TAG fraction of linoleic-type cultivars [3], a ~20-fold difference that explains why sunflower lecithin is oxidatively robust yet nutritionally inert relative to its oil. Saturated acyl chains dominate at 52–58% of PL FA. In high-oleic cultivars, the PL acyl pool shifts toward monounsaturated species (predominantly 18:1), consistent with the cultivar’s substrate-flow patterns; however, total PUFA in PL remains low. This compositional asymmetry is functionally significant: sunflower PL forms ordered, oxidatively stable interfacial layers but offers little nutritional advantage in terms of essential fatty acid delivery, which must be sourced from the TAG fraction [3,8].

### 4.3. Corn

Corn-germ PLs are enriched in PUFA-containing molecular species: in *Zea mays var. Bonus* germ: the dominant PC species are C18:2/C18:1 (*m*/*z* 768, 28.3% of PC) and C18:1/C18:1 (*m*/*z* 770, 40.2%); the dominant PE species is PE-C18:2/C18:2 (*m*/*z* 738, 31.5%) and PE-C18:2/C18:1 (*m*/*z* 740, 40.4%); PI is dominated by PI-C18:2/C18:1 (*m*/*z* 859, 21.4%); PA species are mainly PA-C18:2/C18:2 (*m*/*z* 695, 14.2%) and PA-C16:0/C18:2 (*m*/*z* 671, 35.4%) [9]. The mutation pair *sh1* (shrunken-1) and *wx* (waxy) shifts palmitic-acid glyceride content in storage TAGs but has not been linked to systematic PL acyl-class effects [10].

### 4.4. Pumpkin

Pumpkin PL acyl composition has not been quantified directly. The fatty acid profile of total oil—LA 43–57%, OA 24–34%, palmitic 11–17%, stearic 5–7%, ALA < 0.5%—sets the upper bound on PL composition [12,16,17]. γ-Tocopherol dominates the tocol pool (typically 22–40 mg/100 g oil; in some cultivars up to 40.7 mg/100 g), providing strong interfacial antioxidant protection aligned with the PL fraction [11,17]. The Δ^7^-sterol class characteristic of Cucurbita (spinasterol + Δ^7^-stigmastenol + Δ^7^-avenasterol ≈ 1.1 mg/mL of unsaponifiable matter) is a chemotaxonomic marker; its interaction with PL bilayers differs from the Δ^5^-sterols of other plant oils because of the different position of the steroid-ring double bond, but the membrane biophysical consequences for emulsion stability remain unstudied.

### 4.5. Flax

Flax PLs are extraordinary in their PUFA enrichment: 53.3% of total PL acyl chains are PUFAs, with ALA at 26.7% and LA at 26.6%—values an order of magnitude higher than sunflower, rapeseed or soy PLs (2–4% PUFA) [3]. Saturated acyl chains are restricted to 18% of PL FA (against 52–58% compared to other oils), and MUFA (oleic) accounts for 24.8%, an order of magnitude above sunflower/soy PL, so flax lecithin delivers ALA directly through the PL fraction, not only via TAG. Within molecular species, stearic acid is associated mainly with PC and LPC pools, palmitic with PI, PG, and PA, and LA with PE [18]; the TAG fraction shows comparable enrichment in PUFA molecular species (PLnLn ≈ 5–7%, LLnLn ≈ 12%). This compositional pattern means that flax lecithin delivers ALA directly via the PL fraction, rather than only via TAG-bound ALA—a feature that distinguishes it nutritionally from the other five lecithins.

### 4.6. Camelina

Camelina oil-body PLs reflect the seed’s overall fatty acid profile, in which ALA dominates: 53% of camelina oil-body TAG acyl chains at the sn-2 position are ALA [5]. Direct PC/PE/PI acyl distributions are not published for camelina. The PDCT enzyme catalyzes the bidirectional interconversion of PC and DAG without selectivity between di-oleoyl, di-linoleoyl and di-linolenoyl species, indicating that the PC pool tracks the cumulative fatty acids location through the seed [13]. Engineered DHA1×*fae1* camelina lines accumulate 47.5–58.2% n-3 fatty acids in total seed oil, with DHA reaching 12.6% and total C20^+^ n-3 at 33.0%; in such genotypes, the camelina PL fraction is the most plausible carrier of EPA and DHA among non-marine sources [14].

Across the six oils the phospholipid acyl pool spans an order of magnitude, from strongly PUFA-enriched (flax, 53.3% PL-PUFA, and ALA-rich camelina oil bodies) to strongly PUFA-excluding (sunflower, 2–4%), with hemp and corn intermediate—the property that decides whether a given lecithin is primarily a nutritional or a purely interfacial ingredient.

## 5. Extraction, Degumming, and Their Effect on the Phospholipid Fraction

The same trade-off recurs across all six oils: solvent extraction maximizes phospholipid recovery but mandates aggressive acid/enzymatic degumming, cold pressing preserves co-extracted antioxidants while limiting PL yield, and only aqueous extraction yields native oil bodies—so the processing route, more than the botanical source, sets the final phospholipid load and class balance.

**Figure 5:**
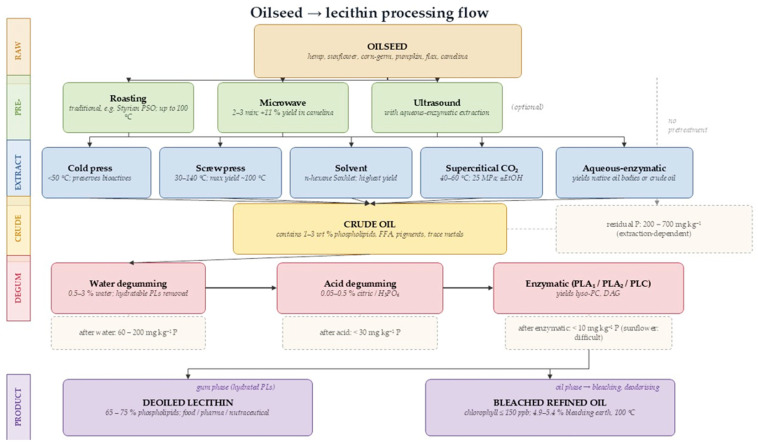
Process flow from oilseed to deoiled lecithin and refined oil. Dashed boxes indicate residual phosphorus targets at each refining stage. Numerical parameters compiled from [1,2,8,20,21,24,25].

The extraction method impacts PL recovery into the crude oil; refining (principally degumming, followed where required by bleaching) determines the final PL content of the food- or pharmaceutical-grade oil. Numerical parameters and quantitative effects are summarized in Table 3, while the general process is presented in Figure 5.

### 5.1. Cold and Screw Pressing

Cold pressing at controlled outlet temperatures (typically <45–50 °C) preserves tocopherols, chlorophyll, carotenoids, and phenolics but yields the lowest PL load relative to solvent extraction. For hemp seed processed at outlet temperatures of 30–140 °C through a screw press, oil yield rises from 21.8% (cold press) to a maximum at 100 °C (oil recovery 66.7%, a 4 percentage-point gain over 30 °C); tocopherols rise from 411 to 513 mg/kg; total phenolics stay constant at 32–42 mg GAE/kg; but peroxide value rises from 6.4 to 13.9 meq O_2_/kg and chlorophyll-a falls from 54.6 to 36.1 mg/kg [21]. Fatty acid composition is invariant across this temperature range. For pumpkin and flax seeds, screw-pressed oils retain the highest content of bioactives (tocopherols, sterols, squalene) but achieve only ~26–39% oil recovery against >50% for hexane extraction [16,17,18]. Thermal pre-treatment of pumpkin seeds prior to pressing modifies oil quality measurably: roasting at 160 °C for 30 min raises peroxide value almost threefold (from 3.82 to 9.89 meq O_2_/kg) and increases carotenoid content fivefold (0.76 to 4.55 mg/kg), but the fatty acid composition remains essentially unchanged across roasting regimes [27]. Thermal pre-treatment simultaneously degrades the oil (peroxides × 3) and concentrates desirable pigments (carotenoids × 5).

### 5.2. Microwave-Assisted and Supercritical CO_2_ Extraction

Microwave pretreatment of hemp seed (1.2 kW, 5 min) raises oil yield without altering fatty acid composition [28]. For camelina, microwave pretreatment of seed pre-adjusted to 2.5% moisture, applied for 3 min, increases cold-press oil yield by ≈11% over the unpretreated control, raises total phenolic compound (caffeic-acid equivalent) to 208 mg/100 g oil, and lowers chlorophyll content; peroxide value remains modest (2.4 meq O_2_/kg under the optimal regime) [25]. Supercritical CO_2_ at 40–60 °C with 0.6–1.5% ethanol cosolvent improves recovery of tocopherols, carotenoids, phenolics, and flavonoids in hemp oil while leaving fatty acid composition intact [24]. For camelina cake, supercritical CO_2_ with 2–30% ethanol cosolvent at 45 °C and 25 MPa recovers up to 84.3% of total PL into the extract, validated by ^31^P-NMR [2]. The highest PL recovery of any green method reviewed, i.e., supercritical CO_2_ can substitute for hexane in lecithin recovery.

### 5.3. Aqueous and Aqueous-Enzymatic Extraction

Ultrasound-assisted aqueous enzymatic extraction of corn germ yields oils of superior antioxidant capacity (DPPH, hydroxyl-radical, and superoxide-anion scavenging) with comparable fatty acid composition to hexane extraction, but with measurable preservation of PLs and tocopherols [10]. Aqueous extraction at moderate pH is also the principal route for recovering intact oil bodies: for camelina, this approach yields the 1.6 µm oleosome dispersion [5]. Aqueous extraction yields lower oil per unit seed than solvent extraction but is the only practical route to native-state oil bodies suitable for direct food use as natural emulsifiers.

### 5.4. Degumming

Three degumming routes are in industrial use [1]. Water degumming exploits the hydration of the more polar PLs (PC, PI, PE) to bring residual phosphorus into the 60–200 mg/kg range; non-hydratable PLs (calcium and magnesium salts of PA, mainly) remain in the oil. Acid degumming adds 0.05–0.5% citric or phosphoric acid (often combined with water) to chelate divalent cations and convert non-hydratable to hydratable PLs, bringing phosphorus to <30 mg/kg. Enzymatic degumming using phospholipases (PLA1, PLA2, or PLC; Lecitase, Purifine, and analogs) hydrolyzes PLs to lysoPLs and free fatty acids (PLA1/PLA2) or to DAG + phosphate ester (PLC). PLC-based degumming brings residual phosphorus below 10 mg/kg only with difficulty in sunflower and other low-PL oils; the limit is set by the resistance of PA to PLC and the slow PLC kinetics on PE [1]. Each successive degumming step lowers residual phosphorus by about an order of magnitude—the staged route to refining-grade oil. For sunflowers specifically, PLA-based enzymatic degumming generates lyso-PC with superior emulsifying properties relative to the parent PC, providing a value-added by-product stream [1,8]. Hempseed oil degumming/neutralization followed by bleaching reduces chlorophyll from 11,400 to 17,500 ppb in crude oil to ≤150 ppb at 4.87% (solvent-extracted) or 5.36% (mechanically pressed) bleaching-earth loadings at 100 °C, 15 min; bleaching also removes residual PL but lowers the polar-antioxidant fraction [20].

### 5.5. Antioxidant Stabilization During Processing

**Figure 6:**
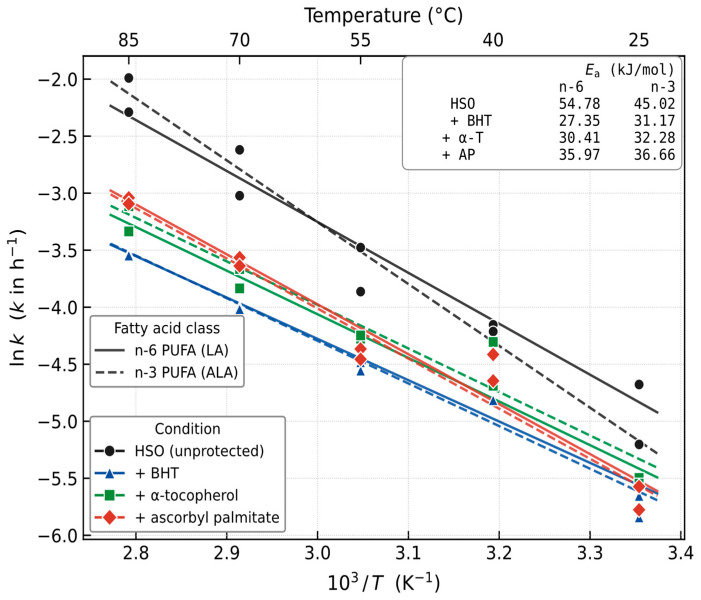
Arrhenius plot of polyunsaturated fatty acid degradation in hempseed oil with and without antioxidant supplementation. The natural logarithm of the first-order rate constant k (h^−1^) is plotted against the reciprocal of absolute temperature (10^3^/T, K^−1^) at five experimental temperatures (25, 40, 55, 70 and 85 °C; equivalent temperatures shown on the upper *x*-axis). Solid lines—n-6 PUFA (linoleic acid, LA), dashed lines—n-3 PUFA (α-linolenic acid, ALA). Four conditions per fatty acid class—unprotected hempseed oil (HSO, black); HSO + butylated hydroxytoluene (BHT, blue); HSO + α-tocopherol (α-T, green); HSO + ascorbyl palmitate (AP, red). Lines are least-squares linear regressions through the measured k values. The inset box lists activation energies (Eₐ, kJ mol^−1^) for each condition. Data points and Eₐ values reproduced from Singh et al. (2020) [26].

Hempseed oil is among the most oxidation-prone of the six. The DT_50_ for n-6 and n-3 PUFAs in crude hempseed oil at 25 °C is c.a.3 and c.a.5 days respectively; at 85 °C the same DT_50_ values fall to c.a.7 and c.a.5 h, with activation energies of 54.8 and 45.0 kJ/mol for n-6 and n-3 PUFA degradation respectively (Figure 6) [26]. Supplementation with α-tocopherol, BHT, or ascorbyl palmitate reduces the PUFA degradation rate constant k by up to 79% and extends the decarboxylation half-life of cannabidiolic acid (CBDA) in the oil from 4 to 17 days at 70–85 °C [26] showing high-PUFA oils have only days of room-temperature stability unprotected and that antioxidants extend this several-fold. These figures set practical limits on the storage stability of high-PUFA, high-PL oils such as hemp, flax, and camelina, and quantify the value of tocopherol-rich co-extractants (γ-tocopherol in pumpkin and camelina, mixed tocols in hemp) as endogenous stabilizers. Co-roasting of oilseeds with antioxidant-rich herbs is an emerging alternative to direct antioxidant addition: roasting pumpkin seeds at 110 °C in the presence of dried marjoram (*Origanum majorana* L.) lowers the peroxide value of the resulting oil by c.a.30% (from 9.17 to 6.44 meq O_2_/kg) and reduces both K232 and K270 extinction coefficients relative to seeds roasted without the herb [27], exploiting the antioxidant terpenes (terpinen-4-ol, γ-terpinene, α-terpinene) that transfer from the herb to the seed matrix during roasting.

## 6. Functional Properties and Applications

### 6.1. Hemp Oil Bodies and Lecithin

Native hemp oil bodies recovered by aqueous extraction are spherical, 3–5 µm in diameter, and present a PC-rich interfacial layer with embedded oleosin-like proteins identified as ≈15 kDa and 25–50 kDa bands by SDS-PAGE [4]. Their isoelectric point is pH 4.0–4.5; below this pH, oil bodies aggregate and the emulsion fails. Above pH 6.5 they are colloidally stable, with PUFA accounting for ≈61% of total acyl chains in the lipid fraction (LA dominant). Hemp lecithin per se is not produced at an industrial scale, but cold-pressed hemp oil rich in tocopherol-stabilized PUFAs is increasingly used in functional food and cosmetic formulations, where its non-psychoactive cannabinoid load (CBDA, CBD; 0.1–1 mg/g typically) adds bioactivity beyond the lipid fraction [15,26].

### 6.2. Sunflower Lecithin in Food Emulsions

Sunflower lecithin is the principal commercial non-GM substitute for soy lecithin and is used in chocolate, bakery, and dairy applications. Recent fractionation work demonstrates that PC-enriched sunflower fractions outperform crude lecithin in ice cream emulsions: the most PC-enriched fraction produced oil droplets of 0.391 µm, zeta potential −28.8 mV, and overrun 46.2%, against 0.547 µm/−24.2 mV/22.7% for crude lecithin [8]. The interfacial salep-PC interaction in the same matrix dominates the rheology, with the consistency coefficient K varying from 3.0 to 52.3 Pa·s^n^ across the formulation series. Hydroxylation of sunflower lecithin further increases hydrophilicity and lowers HLB (hydrophilic-lipophilic balance), expanding the formulation envelope into oil-in-water systems where the parent lecithin underperforms [29].

### 6.3. Corn Germ PL—Bioactive Co-Delivery

Corn germ lipid extracts demonstrate DPPH, hydroxyl-radical, and superoxide-anion scavenging activity attributable in part to the tocopherol and tocotrienol load, which is preserved by ultrasound-assisted aqueous enzymatic extraction [10]. The PC-rich germ PL pool, with its high content of C18:2/C18:1 molecular species, is a candidate carrier for fat-soluble vitamins and for the carotenoid (predominantly β-cryptoxanthin and zeaxanthin) and ferulate fraction of the corn kernel. Corn-fiber gum, a wet-milling co-product carrying 0.24–0.43% bound lipids, behaves as a Pickering-type emulsifier in oil-in-water systems through cooperative hydrophobic anchoring of fatty acid-ferulate-sterol-ester domains [22].

### 6.4. Pumpkin Seed Oil—Δ^7^-Sterol/PL Synergy and Meat Reformulation

Pumpkin seed oil emulsions (with guar gum + inulin, sodium alginate + maltodextrin, etc.) have been used to replace 50 or 100% of pork backfat in deer burgers, raising MUFA + PUFA content and PUFA/SFA ratio while preserving sensory acceptability and reducing lipid oxidation [30,31]. The pumpkin oil PL fraction contributes interfacial stabilization, while the co-extracted Δ^7^-sterols (782–805 mg/100 g oil), squalene (591–747 mg/100 g), and γ-tocopherol (typically 22–40 mg/100 g) deliver an integrated antioxidant matrix. Clinical evidence reviewed by Šamec et al. (2022) supports the use of pumpkin seed oil in benign prostatic hyperplasia (PSO 320 mg/day for ≥3 months reduced IPSS scores), in menopausal cardiovascular indices, and as an adjunct in hypercholesterolemia management; the PL contribution to bioavailability of the active matrix has not been mechanistically isolated [12].

### 6.5. Flax Lecithin—High-PE/High-PUFA Neuromembrane Substrate

Flax lecithin’s exceptional PE content (22.7%) and ALA-rich PL acyl pool make it a candidate substrate for omega-3 delivery via PE—the class preferentially incorporating DHA and arachidonate in mammalian neuronal membranes [2,3]. Flax oil has been incorporated into mayonnaise formulations alongside sunflower and hemp oils, where the PL fraction contributes both nutritional enhancement and emulsion stabilization [18]. The principal processing constraint is oxidative stability: flax oil oxidizes at rates comparable to hempseed oil and benefits from the same antioxidant supplementation strategies [18,26].

### 6.6. Camelina Oil Bodies, Engineered EPA/DHA, and Emerging Applications

Camelina oil bodies (1.6 µm, 0.26% PL, ζ = −40 mV at pH 9) are anionic at neutral pH and aggregate below pH 6.5 [5]. The isoelectric point at pH 3.6 sits below most food-relevant pH ranges, limiting direct use in acidic systems without secondary stabilization. The associated β-sitosterol (2674 mg/kg oil) and γ-tocopherol (670 mg/kg oil) provide endogenous antioxidant protection and a phytosterol delivery channel. Engineered DHA1×*fae1* camelina lines with 33% total C20^+^ n-3 fatty acids [14] offer a route to land-based EPA/DHA production whose lecithin fraction has not yet been compositionally characterized; the PDCT enzyme present in *C. sativa* (which interconverts PC and DAG without acyl-substrate preference) ensures that engineered FA flow through PC into TAG occurs efficiently [13]. Camelina meal, the cake fraction left after oil extraction, contains 40% protein, 17–19% residual fat, and elevated glucosinolates/phytic acid and can be fractionated to upgrade its food-ingredient value [2].

Functionally, the oils map onto their composition: high-PC sunflower and corn lecithins are the strongest interfacial emulsifiers, flax and camelina serve as omega-3 delivery vehicles (PE-bound or within oil bodies), and pumpkin acts as an antioxidant co-delivery matrix—confirming that “superior” is application-specific rather than absolute.

## 7. Limitations and Research Gaps

The revision allowed for the detection of five research gaps, as follows:

1. Because the more polar classes (PC, PI) partition into the aqueous/meal phase more readily than PA, harsher solvent extraction recovers not only more total PL but also a relatively PA-enriched class profile compared with cold pressing; however, class-resolved data by extraction method remains scarce for several of these oils. Class-resolved PL data (PC/PE/PI/PA/lyso percentages) are absent for crude *Cucurbita* and crude camelina oils, leaving total PL data (0.5–1.04% for Styrian pumpkin; 0.26% for camelina oil bodies) without composition context. HPLC-ELSD or ^31^P-NMR characterization of pumpkin and camelina lecithins against the standard soybean/sunflower benchmarks is the highest-priority experimental gap [5,11].
2. The interaction between *Cucurbita* Δ^7^-sterols and PL bilayers—distinct from the well-studied Δ^5^-sterol/PL interactions of most other plant matrices—has not been measured biophysically; this is the principal knowledge gap blocking rational formulation of pumpkin oil emulsions and nanoparticle carriers [11,12].
3. The comparative oxidative stability of high-PUFA PL fractions across hemp, flax, and engineered camelina has been characterized in only one matrix at a time; a uniform-protocol comparison applying Arrhenius kinetics across all three would provide the missing common ground for shelf-life predictions [26].
4. The bioavailability and metabolic fate of PL-bound ALA, EPA, and DHA in human nutrition are quantified by very few clinical studies, leaving open whether PL-bound delivery offers an advantage over TAG-bound delivery for these fatty acids.
5. The regulatory and biochemical roles of PDCT and analogous enzymes in determining seed PL/TAG allocation partitioning—characterized in camelina but not in the other five species—represent the gap between current empirical knowledge and the rational engineering of seed lecithin profiles [13,14].

## 8. Conclusions

**Figure 7:**
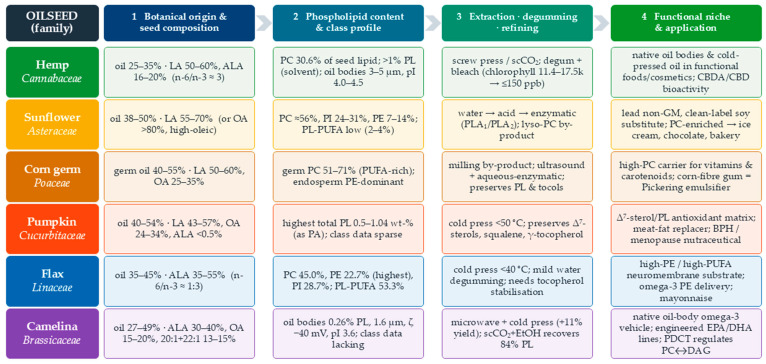
The comparative picture of six oils reviewed. Each oilseed (row) is traced left to right across the four thematic axes—seed composition, phospholipid content and class profile, processing route, and functional niche.

The comparative picture emerges—each oilseed carries a distinctive signature from seed composition, through its phospholipid class profile and the processing route that recovers it, to a specific functional niche (Figure 7)—flax, for instance, runs from a high-ALA seed to a PE-rich phospholipid fraction recovered by gentle cold pressing and delivered as an omega-3 carrier.

Across the six oils reviewed, the total PL fraction varies from 0.26% (camelina oil bodies) to 0.5–1.04 wt.% (Styrian pumpkin oil)—a range that intersects soybean (1.5–3.0%) only at its upper end and that calls into question the assumed similarity of plant lecithins. Sunflower, rapeseed, and (with somewhat lower PC) flax converge on a PC content of 45–56% and PI of 24–32%; the distinctive features are flax’s elevated PE (22.7%) and its order-of-magnitude-higher PL-bound PUFA (53.3%).

Hemp PLs are quantified in tissue context but lack class-resolved oil data; corn-germ PLs are well-characterized at the molecular-species level but their commercial fractionation lags soy and sunflower. Pumpkin oil’s high total PL plus Δ^7^-sterol plus squalene plus γ-tocopherol matrix has been underexploited as a co-delivery system. Camelina oil bodies provide a discrete vehicle for omega-3 delivery (and, in engineered lines, for EPA/DHA) that is structurally distinct from a chemically extracted lecithin and that may bypass the oxidation issues of deoiled lecithin fractions.

Processing constrains every aspect of the resulting lecithin: solvent-extracted oils carry one order of magnitude more PL than mechanically pressed oils, requiring more aggressive acid/enzymatic degumming; cold pressing preserves bioactive co-extracts but limits PL yield; aqueous extraction is the only practical route to native oil bodies; supercritical CO_2_ + ethanol recovers up to 84% of PL with antioxidant activity preserved. The choice of route is not technology-neutral with respect to the final composition. Standardized, class-resolved PL characterization across cultivars and processing conditions—particularly for pumpkin, corn, and camelina—would close the principal data gap blocking the rational use of these six oils as alternatives to soy lecithin in food and pharmaceutical formulations.

## Figures and Tables

**Table 1:** Botanical background and seed composition of the six oilseeds reviewed.

Common Name	Family	Species (Binomial)	Oil Content (% Seed wt)	Protein (% Seed wt)	Dominant Fatty Acid (% TFA)
Hemp	Cannabaceae	*Cannabis sativa* L.	25–35	25–30	LA 50–60; ALA 16–20
Sunflower	Asteraceae	*Helianthus annuus* L.	38–50	20–28	LA 55–70 (or OA > 80, high-oleic)
Maize germ	Poaceae	*Zea mays* L.	40–55 (germ)	18–20 (germ)	LA 50–60; OA 25–35
Pumpkin	Cucurbitaceae	*Cucurbita pepo* L.; *C. maxima* Duch.; *C. moschata* Duch.	40–54	30–40	LA 43–57; OA 24–34; ALA < 0.5
Flax/linseed	Linaceae	*Linum usitatissimum* L.	35–45	20–25	ALA 35–55; LA 14–17
Camelina	Brassicaceae	*Camelina sativa* L. Crantz	27–49	24–31	ALA 30–40; OA 15–20; LA 15–20; 11–20:1 + 22:1: 13–15

OA = oleic acid (18:1 n-9); LA = linoleic acid (18:2 n-6); ALA = α-linolenic acid (18:3 n-3); TFA = total fatty acids. Compiled from [1,3,5,6,8,9,10,11,12,14,15,16,17,18,19].

**Table 2:** Total phospholipid content in the six oils.

Oil/Matrix	Total PL	Other	Source
Hemp (developing seed)	30.6% PC of total lipid; 44.6% galactolipids	LPC + LPE 1.3%; PG highest among PLs in seed	[6]
Sunflower lecithin (HPLC, batch A)	commercial deoiled	balance: lyso + PG	[3]
Sunflower lecithin (HPLC, batch B)	commercial deoiled	balance: lyso + PG	[3]
Corn—germ	major lipid class after TAG	PG detected; *m*/*z* 833 PI species	[9]
Corn—endosperm	major lipid class after TAG	PE-dominant fraction	[9]
Pumpkin (*C. pepo* var. *styriaca*) oil	0.5–1.04 wt.%	PL measured as PA; class data lacking	[11]
Flax lecithin	commercial deoiled	highest PE among compared lecithins	[3]
Camelina oil bodies (aqueous)	0.26% of total lipid	1.6 µm OB diameter; ζ = −40 mV at pH 9; pI 3.6	[5]
Soy lecithin (reference)	commercial deoiled, 65–75% PL	LPC ≈ 8%	[2,3]
Rapeseed lecithin (reference)	commercial deoiled	-	[3]

“dominant” indicates the class is dominant but absolute percentages have not been published. PC—phosphatidylcholine; PE—phosphatidylethanolamine; PI—phosphatidylinositol; PA—phosphatidic acid; PG—phosphatidylglycerol; LPC/LPE—lyso-PC/PE; OB—oil body; pI—isoelectric point, reported precision follows the cited source.

**Table 3:** Extraction and refining parameters and their reported effect on oil yield, phospholipid fraction, and key minor components.

Oil	Method	Process Parameters	Reported Effect	Ref.
Hemp	Screw press, variable T	Outlet 30–140 °C	Yield 21.8% to 21.8% (max 100 °C, +4 pp over 30 °C); tocopherols 411 to 513 mg/kg; PV 6.4 to 13.9 meq O_2_/kg; chlorophyll-a 54.6 to 36.1 mg/kg	[21]
Hemp	Supercritical CO_2_ + EtOH	40–60 °C; 0.6–1.5% EtOH cosolvent	Tocopherol, carotenoid, phenolic, flavonoid recovery rises with EtOH; FA profile unchanged; enzyme-inhibition activity retained	[24]
Hemp (crude to bleached)	Bleaching earth	100 °C, 15 min, 4.9–5.4% earth	Chlorophyll: 11,400 ppb (solv.) or 17,500 ppb (mech.) to ≤150 ppb; β-carotene 60,600 to 6300 ppb (solv.); residual PL removed; polar-antioxidant capacity falls	[20]
Sunflower	Water to acid to PLA1/PLA2 enzymatic	Sequential	Residual P: ≈10 mg/kg achievable with PLA; lyso-PC by-product has higher emulsifying activity than parent PC	[1,8]
Corn germ	Ultrasound + aqueous enzymatic	Cellulase/protease cocktail; sonication	Higher DPPH, OH•, O_2_•^−^ scavenging vs. hexane control; FA profile unchanged; PL and tocopherol better preserved	[10]
Pumpkin	Cold press vs. solvent	Screw press < 50 °C; n-hexane Soxhlet	Yield 26–39% (cold) vs. up to 54% (solvent); cold-press preserves Δ^7^-sterols (782–805 mg/100 g), squalene (591–633 mg/100 g), γ-tocopherol-dominated tocols (94–98 mg/100 g)	[12,16,17]
Flax	Cold press; water degumming	<40 °C press; mild aqueous degumming	Yield 20–30%; preserves PE-rich profile (PC 45.0/PE 22.7/PI 28.7/PA 3.6); high oxidation susceptibility—tocopherol stabilization recommended	[3,18]
Camelina	Microwave pretreatment + cold press	2.5% seed moisture; 3 min MW	Yield +≈11% vs. control; total phenolics 208 mg caffeic acid equiv./100 g; PV 2.4 meq O_2_/kg; lower chlorophyll	[25]
Camelina cake	Supercritical CO_2_ + EtOH	45 °C, 25 MPa, 2–30% EtOH	PL recovery up to 84.3% (^31^P-NMR validated); PC, PE, PI, PS recovered with antioxidative activity preserved	[2]
Hempseed oil (PUFA kinetics)	Antioxidant supplementation	α-tocopherol, BHT, AP at 25–85 °C	DT_50_ n-6/n-3 in unprotected HSO: 3 d/5 d at 25 °C; 7 h/5 h at 85 °C; AOX reduce *k* by up to 79%; CBDA decarboxylation t_1_/_2_ 4 to 17 days with α-tocopherol	[26]

PV = peroxide value; AOX = antioxidant; AP = ascorbyl palmitate; CBDA = cannabidiolic acid; pp = percentage points; DT_50_—time to 50% degradation; reported precision follows the cited source.

## Data Availability

No new data were created.

## Abbreviations

The following abbreviations are used in this manuscript:

ALA	α-linolenic acid (18:3 n-3)
AOX	antioxidant
AP	ascorbyl palmitate
BHT	butylated hydroxytoluene
CBD	cannabidiol
CBDA	cannabidiolic acid
CRISPR-Cas9	clustered regularly interspaced short palindromic repeats-CRISPR-associated protein 9
DAG	diacylglycerol
DGDG	digalactosyldiacylglycerol
DHA	docosahexaenoic acid
DPPH	2,2-diphenyl-1-picrylhydrazyl (radical)
DT_50_	time required for 50% degradation
EPA	eicosapentaenoic acid
EtOH	ethanol
FA	fatty acid
*FAE1*	fatty acid elongase 1
GAE	gallic acid equivalent(s)
GM	genetically modified
HLB	hydrophilic-lipophilic balance
HPLC	high-performance liquid chromatography
HPLC-ELSD	high-performance liquid chromatography with evaporative light-scattering detection
HSO	hempseed oil
IPSS	International Prostate Symptom Score
LA	linoleic acid (18:2 n-6)
LPC	lyso-phosphatidylcholine
LPE	lyso-phosphatidylethanolamine
MGDG	monogalactosyldiacylglycerol
MUFA	monounsaturated fatty acid
MW	microwave
*m*/*z*	mass-to-charge ratio
NADPH	reduced nicotinamide adenine dinucleotide phosphate
OA	oleic acid (18:1 n-9)
OB	oil body (oleosome)
PA	phosphatidic acid
PC	phosphatidylcholine
PDCT	phosphatidylcholine:diacylglycerol cholinephosphotransferase
PE	phosphatidylethanolamine
PG	phosphatidylglycerol
PI	phosphatidylinositol
pI	isoelectric point
PL	phospholipid
PLA_1_/PLA_2_/PLC	phospholipase A_1_/A_2_/C
pp	percentage points
PS	phosphatidylserine
PSO	pumpkin seed oil
PUFA	polyunsaturated fatty acid
PV	peroxide value
SDS-PAGE	sodium dodecyl sulfate-polyacrylamide gel electrophoresis
SFA	saturated fatty acid
TAG	triacylglycerol
TFA	total fatty acids
^31^P-NMR	phosphorus-31 nuclear magnetic resonance spectroscopy
**Symbol**	**Meaning**
Δ^5^/Δ^7^	sterol double-bond position (delta-5/delta-7)
n-3/n-6	omega-3/omega-6 (terminal double-bond position)
Eₐ	activation energy
k	(first-order) rate constant
t_1_/_2_	half-life
K_232_/K_270_	specific extinction coefficient at 232/270 nm
ζ	zeta potential
